# Report of Sociological Committee

**Published:** 1901-11

**Authors:** 


### REPORT OF SOCIOLOGICAL COMMITTEE.

We, your committee, beg leave to state that our field of work is so large and the number of subjects that should be considered by this committee are such that it will require time for due consideration and study.

We have endeavored to arrange a series of papers for this meeting on important subjects, most of which you have heard read we trust with profit.

This, the Tri-State Medical Society representing three States, we hope will unite the medical profession in an effort to secure uniform education and legislation on social questions. It is very difficult for one State alone to make advances on sociological lines without the co-operation of neighboring States.

For instance any restrictions on marriage in one State would not prohibit such parties going to a neighboring State, securing license, getting married, then returning to force their weakened, degenerate offspring on the State that refused them marriage license.

Again in the question of child-labor, if one State passes restrictive legislation, the factories in a neighboring State without restriction can secure cheaper labor, thus working an injury to the mills in a State that is trying to protect the rights, liberty and health of its children. Therefore we strongly urge the necessity of uniformity of action on all social questions in the three States this society represents.

While we as a committee fully recognize that "legislation alone does not completely reform a people," we do feel that judicious legislation with a popular sentiment sufficient to maintain the dignity and enforcement of law is a powerful factor for good, a great educator of the public and a great protection to mankind.

The discussion of such laws by legislative bodies, even if not enacted, are great educational factors.

While it may be true "we cannot make saints by law," it is certain we can to some extent at least, if we so desire, prevent criminals being born by the sanction and license of law, that we can to some extent prevent the propagation of disease and suffering, as well as protect the health, happiness and future of the young of our own land by judicious wholesome legislation.

In order to secure unity of action and uniformity of legislation, we suggest the following, viz.:

1st. That the Tri-State Society recommend the securing as near as possible uniform legislation by the States of Alabama, Georgia and Tennessee on all social questions.

2d. That this society respectfully request the State legislatures of Alabama, Georgia and Tennessee to select three members from the House of Representatives and two from the Senate of each State, to act as a committee on *uniform legislation*, whose duty it shall be to secure as far as possible *uniform laws on such questions as are of common interest to each State, especially laws relating to marriage, divorce, prevention of crime, regulation of child-labor, age of consent, prevention of disease, etc.*

3d. Said committee to meet at the capitol consecutively in each State during session of legislature to consider the question of uniform laws and hear from organizations and any parties interested in such laws.

We recommend that an effort be made to secure enactment of such laws as will prevent the marriage of habitual criminals, the insane, the chronic alcoholic, those suffering with tuberculosis, and the epileptic.

While we recognize the difficulties that will be encountered in the enforcement of such laws, we feel that some steps should be taken to lessen the propagation of crime and disease or at least not give it the sanction and license of the State.

The time has come when the commonwealth should at least not be a party to the development of the criminal and sanction transmission of serious diseased degenerative conditions of the human race.

We, as a State, permit the propagation and development of the criminal and then punish him for being such.

We permit the propagation and development of the insane and diseased in the name of liberty, forgetting or ignoring the fact that the offspring of such have rights and liberties that should not be ignored. The offspring of the criminal, the insane, the alcoholic and the degenerate are born into the world with the marks of crime and degeneration indelibly impressed upon them.

Some are born into a bondage worse even than slavery itself, in this country of freedom where it is said that all men are born equal.

In order that we may enjoy freedom and liberty and be born equal, our parents, grandparents and great-grandparents must be free and equal, not bound and fettered by the iron chain of crime, vice and disease.

If men and women by hereditary transmission and voluntary acts of their own become criminals and degenerates, then they have no moral right to propagate crime and disease and the State has no moral right to license them to do so.

We also recommend that laws be formulated and presented at next meeting of this society for discussion on the rendering of habitual criminals and he who commits rape, sterile by humane surgical methods; also on indeterminate sentences in cases of commitment for crime.

We suggest that in all medico-legal questions involving sanity or insanity, the judge presiding shall appoint, with due provisions for compensation, three competent physicians residing within his jurisdiction to examine accused, whose verdict shall be final as to the *mental responsibility* of person in question.

In order to secure just and proper legislation on the subject of child-labor, we suggest that the cotton manufacturing interests of each State be requested to appoint a delegate or committeeman from each State to work in conjunction with the Sociological Committees from the Tri-State and State Medical Societies of Alabama, Georgia and Tennessee.

For the protection of young girls from the hands of the seducer, we recommend that this society memorialize the State Legislatures of Alabama and Georgia, recommending the raising the age of consent to that of 18 years.

In relation to marriage, we suggest the following as a concise, practical and consistent plan, and recommend the enactment of such laws as will make these requirements effective.

On the back of each license or marriage certificate the following shall be printed for the signature of the male and female applicants:

CERTIFICATE FOR THE CONTRACTING PARTIES.

(Male)—I, ———, living at——— county,——— State, age——, (Female)—I, ———, living at ——— county,——— State, age ——, being duly sworn, certify to the best of my knowledge I am not suffering with and never had any of the following diseases: Insanity, epilepsy, consumption or chronic alcoholism.

Certified by Clerk of Court.

(Signed) —————Male

(Signed) —————Female.

If either applicant has had any of the above diseases, he or she shall then be examined by a reputable and licensed physician, who shall make affidavit that said ——— applicant is free from any of the above diseases.

PHYSICIAN'S AFFIDAVIT.

I, ———, a licensed physician of —— years' practice, residing at ——— county, ——— State, being duly sworm, certify that ———, male applicant, ——— female applicant, for this license to marry, have been thoroughly examined by me, and to the best of my knowledge I find said applicant at this time free from any of the above-named diseases.

Certified by Clerk of Court.

(Signed) —————, M.D.

Note I.—A false affidavit in either of above shall be deemed perjury and punishable as such.

Note II.—If applicant in either case so desires he or she may have medical affidavit made by the President of the Board of Health without expense.

Committee:

R. R. KIME, Chairman.

L. C. LEGRAND, Alabama.

W. G. BOGART, Tennessee.

T. O. POWELL, Georgia.

Report adopted by Tri-State Medical Society at its meeting in Nashville, October 9, 1901.

